# Changes in rhizosphere bacterial communities during remediation of heavy metal-accumulating plants around the Xikuangshan mine in southern China

**DOI:** 10.1038/s41598-018-38360-2

**Published:** 2019-02-13

**Authors:** Dongchu Guo, Zhouzhou Fan, Shuyu Lu, Yongjiao Ma, Xiaohong Nie, Fangping Tong, Xiawei Peng

**Affiliations:** 1 College of Biological Sciences and Biotechnology, Beijing Forestry University, Beijing, 100083 China; 2 Hunan Academy of Forestry, Hunan, 410004 China

## Abstract

Mining and smelting activities are the major sources of antimony (Sb) contamination. The soil around Xikuangshan (XKS), one of the largest Sb mines in the world, has been contaminated with high concentrations of Sb and other associated metals, and has attracted extensive scholarly attention. Phytoremediation is considered a promising method for removing heavy metals, and the diversity and structure of rhizosphere microorganisms may change during the phytoremediation process. The rhizosphere microbiome is involved in soil energy transfer, nutrient cycling, and resistance and detoxification of metal elements. Thus, changes in this microbiome are worthy of investigation using high-throughput sequencing techniques. Our study in Changlongjie and Lianmeng around XKS revealed that microbial diversity indices in the rhizospheres of *Broussonetia papyrifera* and *Ligustrum lucidum* were significantly higher than in bulk soil, indicating that plants affect microbial communities. Additionally, most of the bacteria that were enriched in the rhizosphere belonged to the Proteobacteria, Acidobacteria, Actinobacteria, and Bacteroidetes. In Changlongjie and Lianmeng, the diversity and abundance of the microbial community in the *B*. *papyrifera* rhizosphere were higher than in *L*. *lucidum*. In parallel, the soil pH of the *B*. *papyrifera* rhizosphere increased significantly in acidic soil and decreased significantly in near-neutral soil. Redundancy analyses indicated that pH was likely the main factor affecting the overall bacterial community compositions, followed by moisture content, Sb, arsenic (As), and chromium (Cr).

## Introduction

Antimony (Sb) is a metalloid that is widely used in various industrial products such as batteries, alloys, flame retardants, and catalysts^1^. As a result of the extensive use of Sb and the massive impact of anthropogenic activities (e.g., mining and smelting activities, industrial uses, combustion of fossil fuels, and spent ammunition), large amounts of Sb enter sediments, soils, and water^2^, leading to elevated Sb concentrations. Elevated soil concentrations of Sb reduce crop yields and quality in polluted areas and ultimately affect human health through the soil–crop food chain^3^. Human exposure to Sb damages the liver, lungs, and cardiovascular system^4^. Sb and its compounds are listed as priority pollutants by the U.S. Environmental Protection Agency (USEPA 1979). As the world’s leading producer of Sb, China has accounted for approximately 80% of global annual Sb production during the last decade^2^. The Xikuangshan mine (XKS), located in Lengshuijiang City, Hunan Province, is one of the largest Sb mines in the world^5^. Previous studies have shown that the Sb content of soils around XKS ranges from 101–5045 mg/kg^6^, far higher than the average global soil Sb concentration (approximately 1 mg/kg)^1^ and the average concentration in Chinese soil (approximately 1.06 mg/kg)^7^. However, XKS soil is polluted not only with Sb but also other heavy metals such as arsenic (As), mercury (Hg), cadmium (Cd), chromium (Cr), lead (Pb), and zinc (Zn)^5,8^. Levels of As, Sb, Hg, Cd, Cr, Pb, and Zn have been measured in both soil and plant samples, and the heavy metal concentrations in topsoil are generally higher than background values^5,8^. High levels of toxic metals may pose health risks to humans and plants.

Due to the toxicity and the severity of mine pollution, great attention has been focused on phytoremediation for heavy-metal removal. Plants can extract, transfer, and stabilize various heavy metals, which makes them suitable for removing heavy metals from contaminated environments^9^. *Eichhornnia crassipes* has been reported to be an ideal accumulator of iron (Fe), manganese (Mn), and copper (Cu) from industrial effluents^10^. Yoon *et al*.^11^ showed that several native herbaceous plants had the potential to accumulate Pb, Cu, and Zn at a contaminated Florida site. Chen reported that *Ligustrum lucidum*, *Broussonetia papyrifera*, *Boehmeria nivea*, and *Ailanthus altissima* can be considered accumulators of major heavy metals (Sb, As, Pb, Cd, and As)^12^. Currently, however, full-scale applications of this technique are very limited^13,14^. Soil microbes play an important role in the plant–soil system and can affect the phytoremediation of metal/metalloid-contaminated soils by influencing the bioavailability of metals^15^. A number of microbes, particularly plant-growth-promoting rhizobacteria and arbuscular mycorrhizal fungi (AMF), can enhance the biomass yield and/or heavy metal accumulation of plants^16,17^. As the most abundant group of soil microorganisms, bacteria are actively involved in various biogeochemical processes of the rhizosphere and bulk soils^18^. Compared to bulk soil bacteria, rhizosphere bacteria are more directly beneficial to root patterns and supply nutrient elements to plants^18^. At the same time, rhizosphere microbes are affected by the release of phytochemicals (i.e., root exudates) of plants^19^.

Several studies have investigated microbial communities in Sb mines. Xiao *et al*.^20^ investigated microbial community profiles and their responses to Sb and As pollution in a watershed contaminated by Sb tailing in Guizhou and found that several taxonomic groups were positively correlated with the Sb and As extractable fractions. Another study investigated the abundance and diversity of the bacterial community across different Sb-contaminated soils in XKS and found that the abundance and diversity of the bacterial community varied along a metal contamination gradient^21^. There was even a study on the AMF diversity in the rhizosphere of hyperaccumulators (ramie) in XKS Sb mines^22^; however, it detailed only rhizosphere AMF and ignored rhizosphere bacteria. Few studies have attempted to investigate the diversity of rhizosphere microbial communities of plants in the XKS area.

Based on our previous determination of the enriched heavy metal contents of several plant organs from plants around the XKS Sb mines, we identified four strongly heavy metal-accumulating plants, *L*. *lucidum*, *B*. *papyrifera*, *A*. *altissima*, and *B*. *nivea*^12^. Considering the importance of soil rhizosphere microbes, it is crucial to understand the rhizosphere bacterial diversity and community structure in soils contaminated with heavy metals. We attempted to investigate the changes in the rhizosphere microbial communities of plants in the XKS area. Based on our previous study^12^, we conducted our experiment at the same experimental sites and selected two tree species, *L*. *lucidum* and *B*. *papyrifera*, which are considered to be heavy metal-accumulating plants. The aims of the study were to analyze the changes in each plant’s rhizosphere bacterial community at contaminated sites, to investigate the factors influencing rhizosphere bacterial community structures and diversity, and to unravel the effects of the plants on rhizosphere bacterial communities at XKS. This study will provide a theoretical basis for combined remediation using microbes and plants through the descriptions and evaluations of rhizosphere microbial communities.

## Results

### Soil properties and concentrations of major heavy metals

Soils from different sites varied in their physiochemical properties and heavy metal concentrations, as listed in Tables 1 and 2. For both tree species, the rhizosphere soils generally contained higher moisture content than the bulk soils. The organic matter (OM) of all samples ranged from 1.06% to 5.29%, and the total nitrogen (TN), total phosphorous (TP), and total potassium (TK) were 0.48–1.12, 0.03–0.70, and 9.54–21.02 g/kg, respectively. The soil pH fluctuated widely, from 4.74 (CLR) to 7.49 (LLR). There was a clear difference in pH between the two sites, with acidity at Changlongjie (4.74–5.18 [CBR]) and near-neutral pH at Lianmeng (6.92–7.49 [LBR]). The heavy metal contents of rhizosphere and bulk soils differed. At both sites, lower concentrations of Sb and Cr were measured in the rhizospheres of both tree species compared to the bulk soil samples. The same phenomenon was also observed with Zn and Cd content, except in the bulk and rhizosphere soils of *B*. *papyrifera* at Lianmeng. The As concentration was lower in the rhizosphere of *B*. *papyrifera* than in bulk soil, whereas that in the *L*. *lucidum* rhizosphere was greater than in bulk soil. The Pb concentration displayed no obvious patterns between rhizosphere and bulk soils. Compared to global soil background values, the levels of Sb, As, Cr, and Cd contamination were more severe at our study sites.

**Table 1:** Physicochemical parameters of the soil.

Samples	pH	MoisCont (%)	OM (%)	TN (g/kg)	TP (g/kg)	TK (g/kg)
LLR	7.49 ± 0.09a	28.56 ± 0.83a	4.53 ± 0.79a	0.95 ± 0.012a	0.18 ± 0.030b	16.19 ± 0.315b
LLB	7.43 ± 0.03a	23.53 ± 0.96bc	4.28 ± 0.33a	0.93 ± 0.005a	0.20 ± 0.003b	21.02 ± 0.192a
LBR	6.92 ± 0.08b	24.83 ± 0.12b	3.66 ± 0.18a	0.68 ± 0.014c	0.29 ± 0.003a	11.91 ± 0.298c
LBB	7.34 ± 0.04a	22.24 ± 0.76c	2.61 ± 0.28b	0.78 ± 0.016b	0.30 ± 0.001a	9.54 ± 0.106d
CLR	4.74 ± 0.04b	25.20 ± 0.56a	2.87 ± 0.48b	1.12 ± 0.032a	0.47 ± 0.010a	14.82 ± 0.249b
CLB	4.58 ± 0.02c	23.55 ± 0.37b	2.88 ± 0.11b	0.83 ± 0.003b	0.23 ± 0.015b	10.73 ± 0.128c
CBR	5.18 ± 0.09a	23.77 ± 0.18b	1.06 ± 0.02c	0.48 ± 0.007d	0.70 ± 0.043a	16.10 ± 0.137a
CBB	4.75 ± 0.02b	22.20 ± 0.58c	5.29 ± 0.33a	0.76 ± 0.010c	0.03 ± 0.001b	10.45 ± 0.127c

The first letter in the sample name represents the sampling area; the second represents tree species; the third indicates rhizosphere or bulk soil; C, Changlongjie; L, Lianmeng; L, *Ligustrum lucidum*; B, *Broussonetia papyrifera*; R, rhizosphere; B, bulk soil. Data are reported as means ± standard deviations (n = 3). Different letters indicate significant differences between the four samples at same site (p < 0.05).

**Table 2:** Concentrations of heavy metals in the soil samples.

Samples	Sb (mg/kg)	As (mg/kg)	Pb (mg/kg)	Zn (mg/kg)	Cr (mg/kg)	Cd (mg/kg)
LLR	24.63 ± 1.68b	126.7 ± 34.44a	31.12 ± 1.04a	167.68 ± 15.79b	174.62 ± 2.84b	1.04 ± 0.04b
LLB	117.46 ± 13.81a	88.66 ± 32.08b	41.79 ± 1.45b	223.93 ± 11.63a	205.38 ± 13.95a	2.58 ± 0.12a
LBR	17.36 ± 1.13b	58.78 ± 4.63b	35.84 ± 2.93b	141.62 ± 2.73a	171.40 ± 4.28b	0.54 ± 0.02a
LBB	51.19 ± 6.04a	85.07 ± 0.99a	25.34 ± 1.14a	123.28 ± 5.69b	194.80 ± 4.60a	0.12 ± 0.03b
CLR	20.96 ± 2.49b	165.26 ± 11.53a	33.50 ± 2.20a	206.69 ± 5.64b	291.44 ± 1.75a	0.78 ± 0.06b
CLB	150.18 ± 6.37a	127.47 ± 8.86b	27.83 ± 2.14b	230.07 ± 21.15a	291.96 ± 3.03a	1.27 ± 0.07a
CBR	1.54 ± 0.19b	65.36 ± 2.82b	20.91 ± 1.75b	102.32 ± 12.56b	173.33 ± 14.82b	0.04 ± 0.03b
CBB	32.07 ± 3.93a	130.39 ± 7.13a	31.19 ± 1.98a	152.72 ± 1.95a	240.55 ± 6.41a	0.78 ± 0.08a
Global soil background	1	6	35	90	70	0.35
Multiple	1.54–117.46	9.80–27.54	0.60–1.19	1.14–2.56	2.45–4.17	0.11–7.37

“Multiple” is the heavy metal concentrations in all samples, expressed as a range calculated from the heavy metal content of each sample/Global soil background value. Data are reported as means ± standard deviations (n = 3). Different letters indicate significant difference between the rhizosphere and bulk soil at the 0.05 level.

Note: The English in this document has been checked by at least two professional editors, both native speakers of English. For a certificate, please see: http://www.textcheck.com/certificate/606Jrh.

### Taxonomic distribution and comparisons between the two sites

The rarefaction curves for bacterial communities indicated that variation in operational taxonomic unit (OTU) density within the soil samples was sufficiently captured at the sequencing depth used (Fig. S1). Therefore, the data were sufficient to allow analyses of microbial communities. There were 609,268 valid reads identified for all soil samples collected (including repetition) through the Illumina MiSeq platform after filtering out the low-quality reads and chimeras, and trimming the adapters, barcodes, and primers (Table S2). These reads were clustered into 3,285 bacterial OTUs and 37 bacterial phyla, whereas only 16 phyla presented a sum abundance greater than 99% and approximately 1% of the sequences remained unidentified.

**Figure 1:**
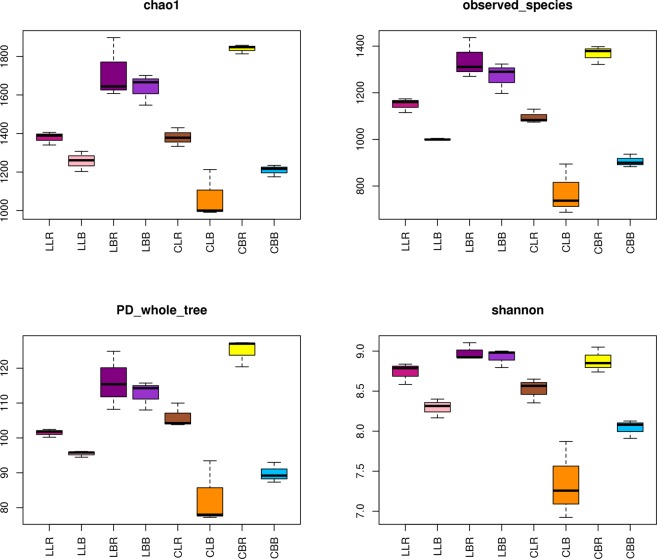
Number of sequences analyzed and diversity/richness indices of the 16S rRNA bacterial libraries obtained from clustering at 97% identity.

The soil bacterial diversity and abundance were evaluated in the samples by estimating OTUs at a 3% sequence dissimilarity level. The Chao 1 index was used as an indicator of species richness (Fig. 1 and Table S1). The PD_whole-tree index was used as an indicator of phylogenetic diversity. The Shannon and observed_species indices were used to show the diversity of the microbial community in each sample. The results indicated that bacterial richness and diversity differed between the two sites. There were also differences between the rhizosphere and bulk soils of each tree species, except in the LBR and LBB samples. Notably, the differences between the rhizosphere and bulk soil samples of the tree species were greater at Changlongjie than at Lianmeng. The CBR sample showed the highest overall bacterial diversity and richness. The above results indicate that soil biodiversity differed between sampling locations, and different soil properties and tree species had an effect on bacterial diversity and richness.

**Figure 2:**
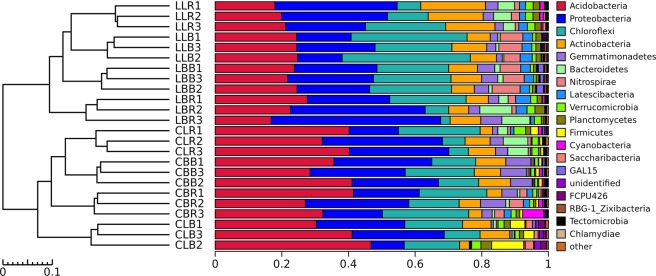
(Left) The UPGMA tree showing clusters of bacterial communities based on weighted UniFrac with 100% support at all nodes. (Right) The bacterial communities and their diversity at the phylum level in all soil samples. Samples were named with letters indicating their collection location (L, Lianmeng; C, Changlongjie), tree species (L, *L*. *lucidum*; B, *B*. *papyrifera*), substrate (R, rhizosphere; B, bulk soil), and number of replications (1–3).

The bacterial community composition of each sample at the phylum level is shown on the right side of Fig. 2. The dominant taxa across all soil samples were Acidobacteria, Proteobacteria, Chloroflexi, Actinobacteria, and Gemmatimonadetes; these groups accounted for more than 80% of the bacterial phyla sequenced. The relative abundance of each bacterial phylum indicates that each bacterial group responded to differences between the sites. The differences between the two sites can be seen in Figs 1 and S2. More Proteobacteria, Chloroflexi, Actinobacteria, and Nitrospirae were detected in Lianmeng samples than in Changlongjie samples. By comparison, Acidobacteria were relatively more abundant in Changlongjie samples than in Lianmeng soil samples. This may be closely related to the acidic environment in the Changlongjie soils^23^.

The cluster tree on the left side of Fig. 2 is based on the Bray-Curtis distances. The branches indicate the distances of the genetic relationships among all samples. The bacterial community structure was classified into two large groups with good similarity between parallel samples. The first group was composed of the samples from Lianmeng, with a pH range of 6.92–7.49. The second group was composed of samples from Changlongjie, with a pH range of 4.58–5.18. The bacterial communities at the two sites were clearly separated, likely due to the difference in pH.

### Bulk soils vs. rhizospheres of the different plant species at each site

**Figure 3:**
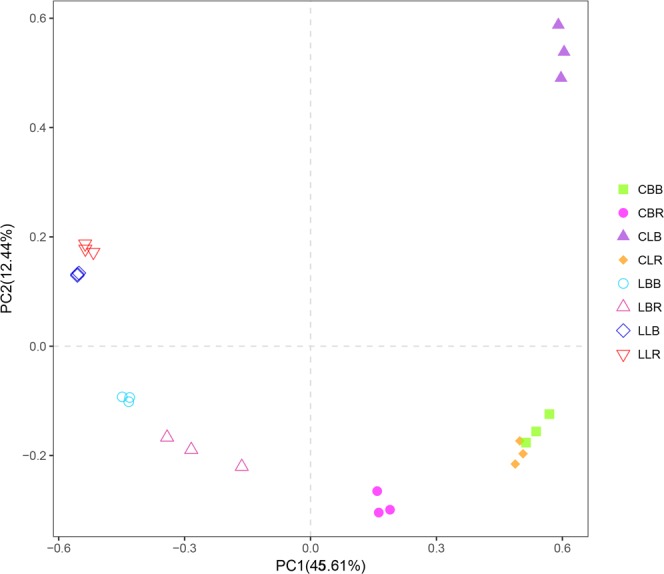
Principal component analysis of bacterial 16S rDNA sequences of all samples based on total OTU level.

Principal component analysis (PCA) was used to visualize differences in community structure between rhizosphere and bulk soil samples (Fig. 3). Principal components 1 and 2 explained 45.61% and 12.44% of the total sample variability, respectively. The results indicated a relatively clear separation between the samples from the two sites. However, compared to those at Changlongjie, the rhizosphere and bulk soil samples of both tree species at Lianmeng were closer together on the PCA plot. This is may be due to the difference in pH values between the two sites. The analysis revealed clear differences between the bacterial community structures of rhizospheres and bulk soils.

**Figure 4:**
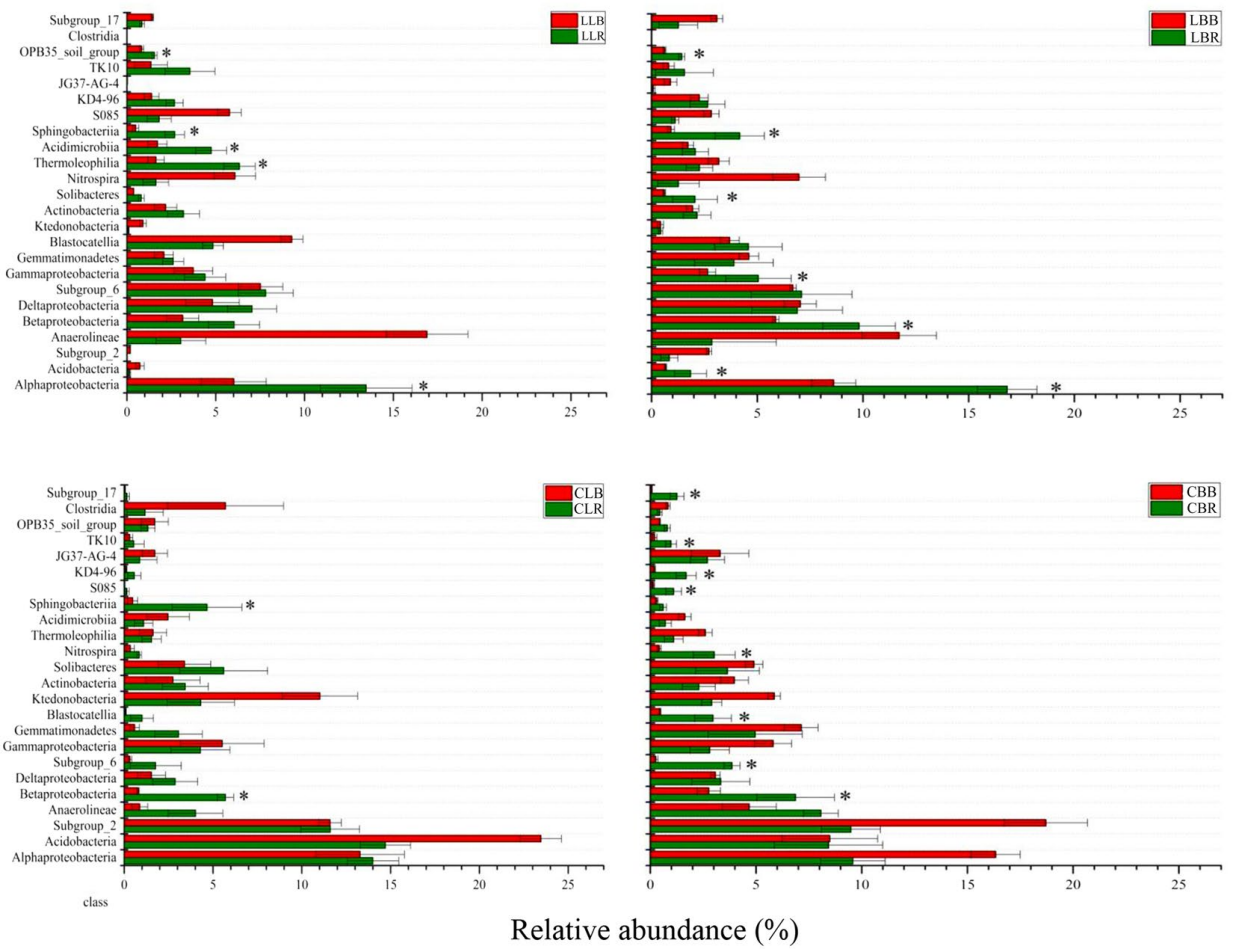
Comparison of the relative bacterial abundance in rhizospheres and bulk soils. The error bars show the calculated standard deviation in triplicate samples and asterisks (*) indicate categories significantly more abundant in the rhizosphere (*P* < 0.05).

For each site, significance analyses of the bacterial community were conducted to determine the selective/enrichment effects of the rhizosphere in each tree stand (Fig. 4). These analyses were performed at the class level because the bacteria were not fully identified at other levels. We selected the bacterial taxa with an average abundance ≥1% for analyses using ANOVA (*P* > 0.05 for each taxon tested). At the Lianmeng site, the rhizosphere samples of *L*. *lucidum* were significantly enriched in Alphaproteobacteria, Thermoleophilia, Acidimicrobiia, Sphingobacteria, and OPB35_soil_group. The *B*. *papyrifera* rhizosphere samples were significantly enriched in Alphaproteobacteria, Acidobacteria, Betaproteobacteria, Gammaproteobacteria, Solibacteres, Sphingobacteria, and OPB35_soil_group. The rhizospheres of both plants were significantly enriched in Alphaproteobacteria, Sphingobacteria, OPB35_soil_group, and Betaproteobacteria. At the Changlongjie site, the *L*. *lucidum* rhizosphere samples were significantly enriched in Betaproteobacteria and Sphingobacteria. The *B*. *papyrifera* rhizosphere samples were significantly enriched in Betaproteobacteria, Subgroup_6, Blastocatellia, Nitrospira, S085, KD4–96, TK10, and Subgroup_17. The rhizospheres of both plants at Changlongjie were significantly enriched in only Betaproteobacteria.

### Relationships between bacterial communities and environmental variables

**Figure 5:**
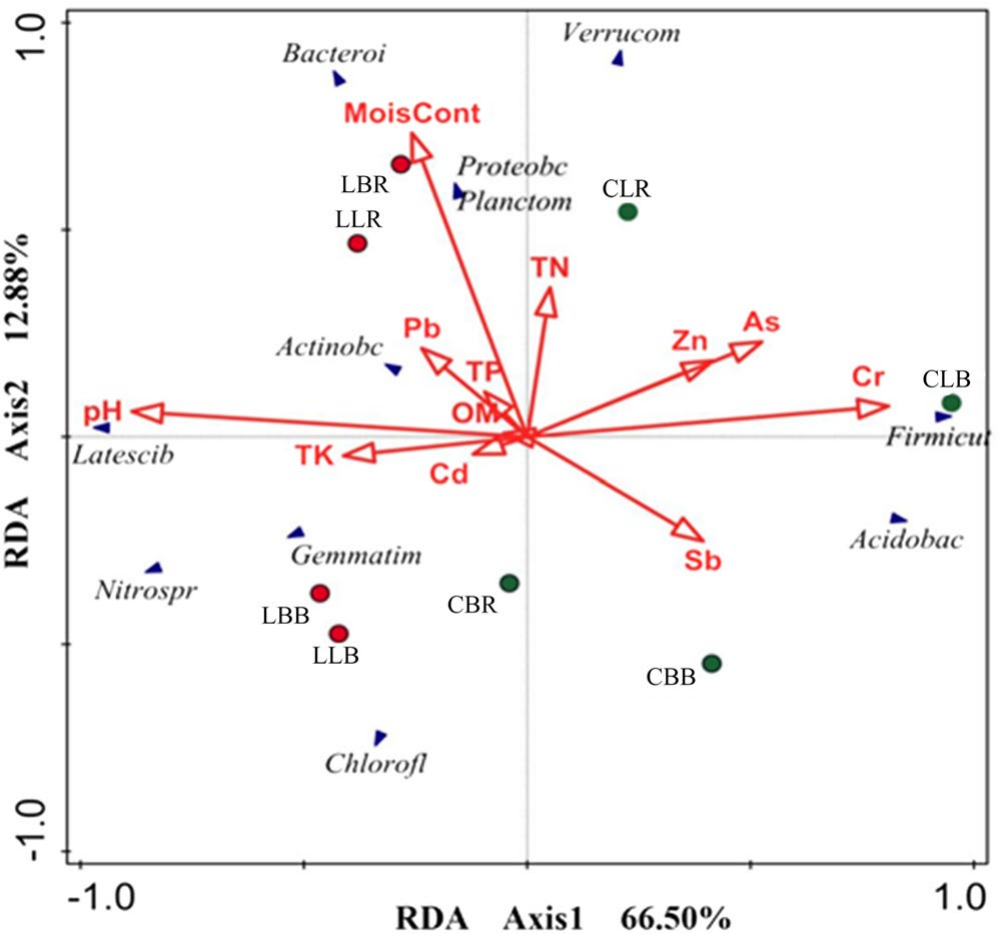
Redundancy analysis ordination diagram of bacteria in relation to measured physicochemical parameters and major heavy metals for all samples.

Redundancy analysis (RDA) showed that the relative abundance of bacteria was affected by both growing position and soil properties (Fig. 5). The first two axes explained 66.50% and 12.88% of the total variance, respectively. The strongest determinant of bacterial communities was pH, followed by Cr, Sb, As, Zn, and moisture content. The samples at Lianmeng were positively correlated with pH, whereas the samples from Changlongjie, except for CBR, were negatively correlated with pH. The first component of the RDA (RDA1) separated Lianmeng soil samples from the four Changlongjie soil samples. These results imply a clear difference in bacterial abundance at the two sites. In addition, the bacterial abundance of the three rhizosphere samples (LLR, LBR, and CLR) was positively correlated with moisture content, whereas the distribution in bulk soils was relatively dispersed. Separately, the abundance in Lianmeng samples was positively related to pH and moisture content, and the abundance in Changlongjie samples was positively related to Cr, Sb, and As. Thus, ecological habitat had a significant effect on bacterial abundance. Soil pH had a positive effect on Latescibacteria and a negative effect on Acidobacteria and Firmicutes. Soil moisture had a positive effect on Proteobacteria, Bacteroidetes, and Planctomycetes. Soil Sb had a negative effect on Actinobacteria. As and Zn had negative effects on Gemmatimonadetes and Nitrospirae.

## Discussion

Soil pollution with Sb from mining and manufacturing has become increasingly severe^6^. The pollution from the XKS Sb mine is due not only to Sb, but also As, Cd, Cr, Pb, and other heavy metals. Compared to traditional methods of soil remediation for heavy metals, such as physical and chemical approaches, phytoremediation is considered effective and more environmentally friendly^24^. In this study, successful plant establishment was observed in the XKS area after ecological restoration for more than 10 years. The plant species had diverse effects in altering the soil properties and heavy metal concentrations. With phytoremediation practice, the microbial diversity in the rhizosphere was remarkably changed.

We investigated the composition and diversity of the bacterial communities of *L*. *lucidum* and *B*. *papyrifera* at two different sites. We observed clear differences between the two sites as well as differences between bulk soil and rhizosphere samples (Fig. 3), indicating that changes in the bacterial community structure were related to plant species and soil characteristics^25^. By comparing the bacterial communities in bulk soils or rhizospheres at the phylum level for the same tree species at two sites, we demonstrated that these communities were impacted by their ecological habitats. For example, Acidobacteria communities were more abundant in *L*. *lucidum* rhizospheres at Changlongjie than at Lianmeng, and Nitrospirae communities were more abundant in *B*. *papyrifera* bulk soils at Lianmeng than at Changlongjie. RDA revealed that bacterial community structures were influenced by pH, Cr, Sb, As, Zn, and moisture content, but pH was the dominant factor. Previous studies have shown that pH is a key factor in the distribution of bacterial populations^26,27^ and bacterial community diversity is highest in soils with near-neutral pH and lowest in soils with pH < 5^21^. In this study, pH values differed between Changlongjie (4.74–5.1) and Lianmeng (6.92–7.49). It has been suggested that pH affects bacterial survival directly and/or by controlling ancillary environmental parameters that are closely related to soil pH, such as cationic metal solubility^28^. Many bacteria have near-neutral intracellular pH levels, and their growth is sustainable over a relatively narrow pH range (3–4 pH units)^29^. Indeed, pH had a significant effect on the bacterial communities in this study (Fig. 5). The effects of Cr on the bacterial communities at XKS were greater than the effects of Sb and As, which has not been reported in previous studies on the structure of XKS microbial communities. One potential explanation is that the metal speciation of Cr affected the organic acid exudation rate and the composition of root exudates, and the root exudates greatly affected the microbial activity and bacterial communities in the soil^30,31^.

Plant species, as well as ecological habitats, have a substantial influence on the structure of rhizosphere-associated microbial populations. The influence of plant species has been clearly shown in several studies. Kowalchuk reported that plant species impact the rhizosphere bacterial communities of *Cynoglossum officinale* and *Cirsium vulgare*^32^. Uroz *et al*.^33^ also reported the effects of plant species on rhizosphere bacterial communities. The rhizosphere, a unique microenvironment in terrestrial ecosystems, is described as the portion of the soil in which microorganism-mediated processes are under the influence of the root system^19^. The biomass and activity of microorganisms are higher in the rhizosphere than in bulk soils, owing to the exudation of compounds by plant roots^34^. Indeed, in our study, the diversity (Shannon index) and abundance (Chao1 index) of rhizosphere bacterial communities under each tree species were greater than in the bulk soils (Fig. 1), indicating that the trees influence the structure and diversity of the microbial community. Sun *et al*.^35^ also reported similar results in studying changes in the rhizosphere microbial communities of *Pennisetum purpureum*, *Typha angustifolia*, and *Alnus cremastogyne* in copper mine tailings in China.

In parallel, Hinsinger *et al*.^36^ reported that plants can regulate rhizosphere pH, and a major process contributing to root-induced pH changes in the rhizosphere is the release of charges carried by H^+^ or OH^−^. The root-mediated pH changes in the rhizosphere depend on constraints from the enviroment^37^. In this study, we found that the soil pH in the rhizosphere of *B*. *papyrifera* was obviously elevated compared to bulk soil in Changlongjie, whereas *B*. *papyrifera* rhizosphere pH was significantly reduced in Lianmeng. These pH changes were not observed in *L*. *lucidum*. The results indicate that *B*. *papyrifera* regulates the rhizosphere soil pH better than *L*. *lucidum*. These root-mediated pH changes affect the bioavailability of many nutrients and toxic elements and improve the growth environment of rhizosphere microorganisms. This indicates that *B*. *papyrifera* can create better survival conditions for itself and its rhizospheric microbes, in addition to playing a more significant role in the restoration of heavy metal-contaminated soil.

Each plant species is thought to select specific microbial populations^38^. The compartment effect (i.e., rhizosphere vs. bulk soil) was used to determine the selective effects of the rhizosphere habitat on bacteria (Fig. 4). The sequences generated from the Lianmeng and Changlongjie soil samples were analyzed independently. The rhizospheres of both tree species at Lianmeng were enriched in Alphaproteobacteria, Sphingobacteria, and OPB35_soil_group, whereas the other bacteria enriched in the rhizosphere differed between the two species (Fig. 4). At Changlongjie, the rhizospheres of both tree species were enriched in Betaproteobacteria only. This indicates that the selection of rhizosphere microflora by these two tree species is different. Uroz *et al*.^33^ reported the same results when comparing the rhizosphere selectivity of beech and Norway spruce trees.

Notably, most bacteria enriched in the rhizosphere belonged to the Proteobacteria, Acidobacteria, Actinobacteria, and Bacteroidetes. Researchers have repeatedly demonstrated that Proteobacteria may be the most metal-tolerant organisms found at metal-contaminated sites^39,40^. Their ecological and metabolic abilities have been reported to be adaptable to the extreme environment of mine tailings and to reduce the toxicity of heavy metals^41,42^. Acidobacteria are disintegrators of organic matter and are involved resilience in nutrient-deficient environments^43^. At the same time, Actinobacteria are related to defense against soil diseases and improvement of root nodulation efficiency^44^. Bacteroidetes are involved in the degradation of high-molecular-weight organic matter in the rhizosphere^45^. These rhizospheric microorganisms are involved in the toxicity reduction of heavy metals in soil, the prevention and control of soil diseases, the improvement of root nodulation efficiency, and adaptation to the extreme environment of mine tailings. The resistance of plants can be promoted in extreme environments, which will be helpful for phytoremediation. Therefore, we can artificially add microbes that are enriched in the rhizosphere to the rhizospheres of other plants, which will promote plant repair. This has been done previously in heavy metal-contaminated rice fields^46^.

The effects of heavy metals on bacterial communities cannot be ignored. Cr, Sb, and As affected the distribution of bacterial communities, and the levels of each differed between the plant rhizospheres and bulk soils. At both sites, Sb and Cr levels were lower in the rhizospheres of both tree species than in bulk soils, whereas As content was greater in the rhizosphere of *B*. *papyrifera* than in bulk soil, but lower in the rhizosphere of *L*. *lucidum* than in bulk soil. The diversity of the bacterial community was somewhat greater in the *B*. *papyrifera* rhizosphere than in that of *L*. *lucidum* (Fig. 1). The bacterial diversity index is negatively correlated with heavy metals in forest soils^47^. Due to the decreased concentrations of heavy metals in the rhizosphere, the bacterial diversity may showed an obvious increasing trend^48^.

Chen^12^ demonstrated that the two tree species can be used as heavy metal-accumulating plants at the two sites; thus, the reduction in heavy metals in the rhizosphere may be due to absorption by plants.

This study considered the effects of tree species and XKS sites on bacterial communities. We concluded that the bacterial community structure was influenced by both ecological habitat and tree species. However, the effects of each on bacterial communities differed between acidic and near-neutral soils. In addition, *B*. *papyrifera* may serve as a better accumulator plant than *L*. *lucidum* at Changlongjie. This speculation is based on the evidence that the bacterial diversity of the *B*. *papyrifera* rhizosphere was greater than that of the *L*. *lucidum* rhizosphere, and levels of Sb, As, and Cr in *B*. *papyrifera* rhizospheres were lower than those in bulk soil, although there was a similar trend in Sb and Cr, but not As, in the *L*. *lucidum* rhizosphere. However, this is only a preliminary evaluation based on bacterial community structure and heavy metal content. Further research elucidating the mitigation effects of the tree species and the functions of the bacteria that were significantly enriched in the rhizosphere is needed.

## Conclusions

We observed the rhizosphere bacterial communities of heavy metal-accumulating plants at two sites near XKS in Lengshuijiang City, Hunan Province. By analyzing the changes of plant rhizosphere microflora in two regions, we found that plant species and ecological habitats cooperatively shaped the structure of microbial communities in the rhizosphere. In particular, *B*. *papyrifera* modified the soil pH and several heavy metal levels in the rhizosphere. Also, the diversity and abundance of the microbial community in the *B*. *papyrifera* rhizosphere was higher than in bulk soil and other plants. Therefore, we hypothesize that *B*. *papyrifera* may be more suitable for phytoremediation than *L*. *lucidum* at these two sites. We also made a preliminary assessment of the significant rhizobacterial communities of each tree species at the two sites to provide a theoretical basis for joint restoration using plants and microbiota. Most bacteria enriched in the rhizosphere belonged to Proteobacteria, Acidobacteria, Actinobacteria, and Bacteroidetes. These bacterial groups have been reported to be associated with heavy metal resistance, decomposition of organic matter, enhancement of root nodulation efficiency, and adaptation to extreme environments. In future studies, we should attempt to isolate these bacteria and explore their functions in phytoremediation. This will provide a theoretical basis for combined remediation using microbes and plants. Finally, we found that the effects of Cr on the bacterial communities at XKS were greater than the effects of Sb and As, which has not been reported in previous studies.

## Materials and Methods

### Site location and sample collection

**Figure 6:**
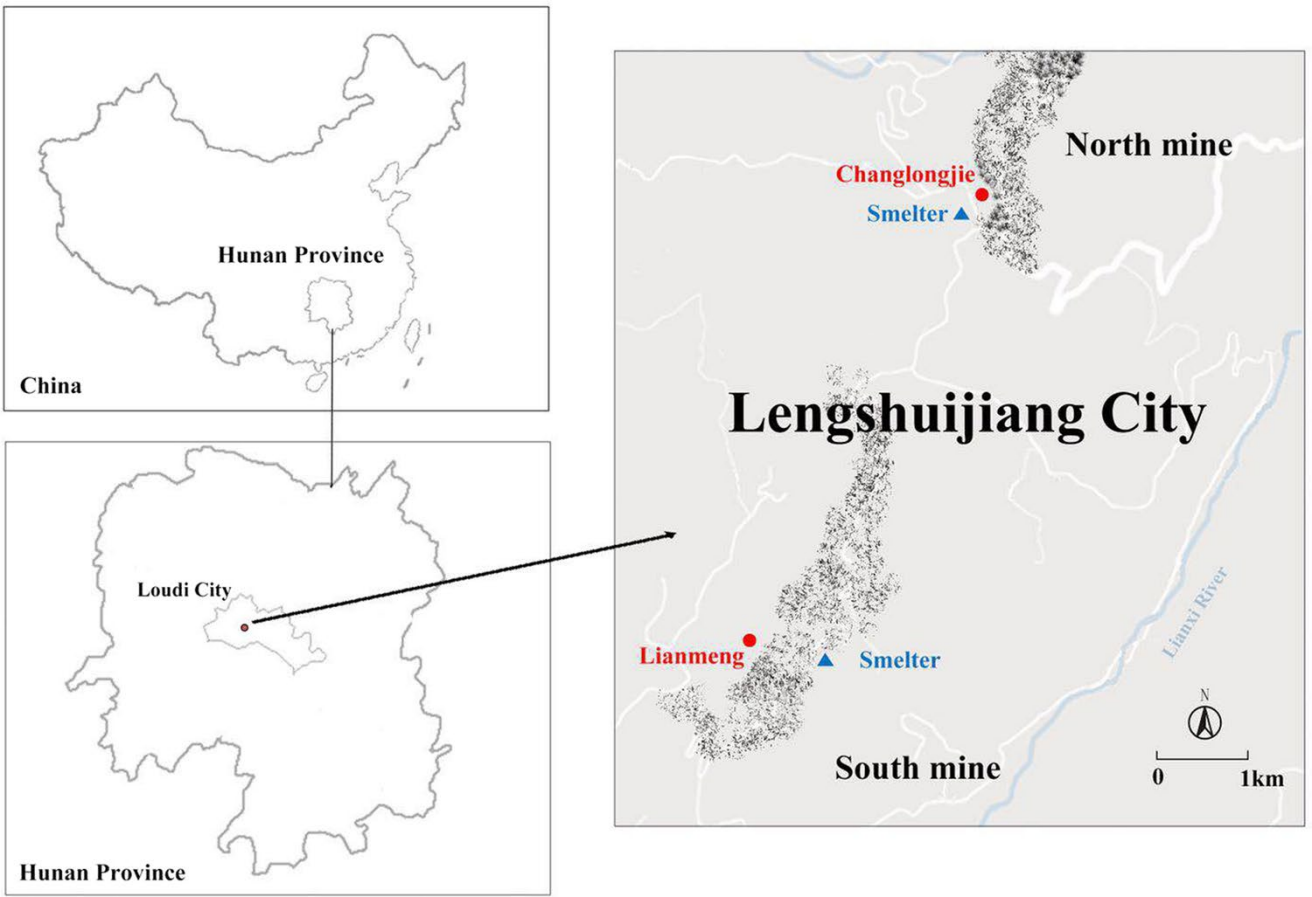
Location of the two sampling sites of soil samples around XKS of China. Map in this figure was generated by sofware Photoshop cc2015.

The study site is located at the XKS Sb mine, Lengshuijiang City (27°30′49″–27°50′38″N, 111°18′57″–111°36′40″E), Hunan Province, Southwest China (Fig. 6, generated by Photoshop CC 2015). The mine is one of the world’s major producers of Sb. It is renowned as the “world’s antimony capital”. In this region, mining and smelting activities have resulted in the pollution of soil, water, and sediments with Sb and other metals over the past approximately 100 years^2^. In November 2015, rhizosphere and bulk soil samples from *L*. *lucidum* and *B*. *papyrifera* were collected from two sites near the XKS mine, Lianmeng and Changlongjie. The rhizosphere soil samples were collected from soils adhering to plant roots (ca. 1–2 mm to roots) from different locations within each plant stand and systematically pooled together. Bulk soil ~50 cm from the trees was collected using a soil corer at soil depths of 0–20 cm. Each sample was randomly collected using the five-point sampling method, then pooled together into one composite sample. We set up three plots (20 × 20 m) for each tree species at each site. We took one rhizosphere and one bulk soil sample from each plot; the three soil plots were three repetitions of one sample. A total of 24 composite samples were collected (two tree species × three replicates × two sites × [rhizosphere + bulk soil]). Samples were encoded with letters indicating their collection location (L, Lianmeng; C, Changlongjie), tree species (L, *L*. *lucidum*; B, *B*. *papyrifera*), and substrate (R, rhizosphere; B, bulk soil).

Each soil sample was collected and passed through a 2-mm sieve, then stored in a sterile polyethylene bag at 4 °C for measuring physicochemical parameters, and at −20 °C for DNA extraction.

### Physicochemical analyses of soil and determination of target metals

Soil moisture content, pH, organic matter (OM), total nitrogen (TN), total phosphorus (TP), and total potassium (TK) were assessed. Moisture was estimated using the oven dry-weight method. Soil pH was measured using a glass electrode pH meter (Sartorius PB-10; Sartorius Scientific Instruments Co., Ltd., Beijing, China) in a suspension of 1 g soil in 5 mL distilled water. OM content was determined by the Walkey and Black method^49^. The determination of TN, TP, and TK were carried out in accordance with the national standards of the People’s Republic of China. Briefly, 0.2 g of soil was added to 5 ml of perchloric acid and 5 ml of hydrofluoric acid for low-temperature digestion, followed by determination of TP and TK by the Mo-Sb colorimetric method and the flame photometer method, respectively. TN was extracted from 0.2 g of soil by digestion with 5 ml of concentrated sulfuric acid, and then determined by the Kjeldahl method. According to Wilson^50^, heavy metal contaminated soil was analyzed by digestion of 0.5 g of soil with 10 mL of concentrated HNO_3_, following the microwave-nitric acid method. The concentrations of Sb, As, Pb, Zn, Cr, and Cd in the soil samples were measured using inductively coupled plasma optical emission spectrometry.

### High-throughput sequencing of the V3–V4 regions of 16S rRNA genes

Total genomic DNA was extracted from each soil sample using a FastDNA spin kit (MP Biomedicals, Santa Ana, CA, USA) following the manufacturer’s protocol. The concentration and purity of the extracted DNA were confirmed using 1% agarose gel electrophoresis.

The hypervariable V3–V4 region of the bacteria 16S rRNA gene was amplified using the 338 f/806r primer set (338 f: 5′-ACTCCTACGGGAGGCAGCA-3′, 806r: 5′-GGACTACHVGGGTWTCTAAT-3′)^51^. These primers contain a set of 8-nucleotide sequences unique to each sample. The PCR program was as follows: a 5-min initial denaturation at 95 °C, 25 cycles at 95 °C for 30 s, 55 °C for 30 s, and 72 °C for 30 s, with a final extension at 72 °C for 10 min. PCR reactions were performed in triplicate. The high-throughput sequencing of the 16S rRNA amplicons was carried out on an Illumina Miseq PE300 sequencing platform (Illumina, Inc., San Diego, CA, USA) at Allwegene Tech, Ltd. (Beijing, China). All datasets were analyzed using QIIME, based on sequence length, quality, primers, and tags; the raw sequences were selected and the low-quality sequences were removed^52^. High-quality sequences were trimmed using the Illumina Analysis Pipeline version 2.6. The SILVA Classifier tool was used to classify the unique sequence set into operational taxonomic units (OTUs)^53^, with a threshold of 97% identity^54^.

### Data analyses

All sequences were analyzed using the bioinformatics platform Mothur (v.1.33.0)^55^. The similarity of bacterial communities among different soil samples was determined using both weighted and unweighted UniFrac. PCA based on weighted UniFrac distances was performed to quantify differences in community composition at the OTU level. Rarefaction analyses were used to measure whether sequences sampled were sufficient to capture the total richness at a genetic distance of 0.03. Shannon, Chao1, observed_species, and the PD_whole-tree indices were determined for the diversity and richness of the bacterial community. RDA was used to identify the effects of environmental factors on bacterial communities, based on a relative abundance greater than 1% of bacterial communities at the phylum level, with Canoco 5.0 software. Before we did the RDA analysis, we unified the units of various environmental factors. The effects of tree species on the abundance of the different taxa at the class level were tested using analysis of variance (ANOVA) at a threshold level of *P* = 0.05 using IBM SPSS Statistics v.19.0 (IBM Corp., Armonk, NY, USA).

## Supplementary information

Supplementary Information
