# Supplementary material for: Changes in rhizosphere bacterial communities during remediation of heavy metal-accumulating plants around the Xikuangshan mine in southern China
Source: Sci Rep. 2019 Feb 13;9:1947. doi: 10.1038/s41598-018-38360-2 (PMC6374380; doi:10.1038/s41598-018-38360-2)
Supplement: Supplementary file 1 — Supplementary Information [file 41598_2018_38360_MOESM1_ESM.doc]

**Supplementary** **Information**

**Changes in rhizosphere bacterial communities during remediation of** **heavy metal-accumulating plants around the Xikuangshan mine in southern China**

**Dongchu Guo ^1^, Zhouzhou Fan** **^1^, Shuyu Lu ^1^, Yongjiao Ma ^1^, Xiaohong Nie ^1^, Fangping Tong ^2^,
Xiawei Peng^*^**

^1^College of Biological Sciences and Biotechnology, Beijing Forestry University, Beijing, 100083, China.

^2^Hunan Academy of Forestry, Hunan, 410004, China.

**Dongchu Guo and Zhouzhou Fan contributed equally to this work. Correspondence and requests for materials should be addressed to Xiawei Peng (email: xiaweipeng@163.com)**

| **SampleID** | **chao1** | **goods_coverage** | **observed_species** | **PD_whole_tree** | **shannon** |
| --- | --- | --- | --- | --- | --- |
| LLR1 | 1405.53 | 0.97 | 1173.80 | 101.79 | 8.79 |
| LLR2 | 1388.70 | 0.98 | 1160.00 | 102.43 | 8.84 |
| LLR3 | 1340.31 | 0.98 | 1114.80 | 100.19 | 8.58 |
| LLB1 | 1261.02 | 0.98 | 999.30 | 95.82 | 8.31 |
| LLB2 | 1307.45 | 0.97 | 998.70 | 94.47 | 8.17 |
| LLB3 | 1203.11 | 0.98 | 1004.40 | 96.12 | 8.40 |
| LBR1 | 1897.58 | 0.96 | 1436.50 | 124.85 | 8.92 |
| LBR2 | 1607.39 | 0.97 | 1311.10 | 115.41 | 9.10 |
| LBR3 | 1644.50 | 0.97 | 1270.30 | 108.19 | 8.92 |
| LBB1 | 1665.93 | 0.97 | 1290.00 | 114.27 | 8.98 |
| LBB2 | 1547.28 | 0.97 | 1197.00 | 108.02 | 8.79 |
| LBB3 | 1700.91 | 0.96 | 1323.10 | 115.74 | 9.00 |
| CLR1 | 1429.83 | 0.97 | 1129.60 | 109.99 | 8.65 |
| CLR2 | 1333.22 | 0.97 | 1083.00 | 104.24 | 8.57 |
| CLR3 | 1378.09 | 0.97 | 1074.20 | 103.80 | 8.35 |
| CLB1 | 1213.12 | 0.97 | 894.30 | 93.44 | 7.87 |
| CLB2 | 991.30 | 0.98 | 687.50 | 77.21 | 6.92 |
| CLB3 | 999.89 | 0.98 | 737.20 | 77.98 | 7.26 |
| CBR1 | 1813.32 | 0.96 | 1321.60 | 120.40 | 8.74 |
| CBR2 | 1857.56 | 0.96 | 1397.60 | 127.30 | 9.05 |
| CBR3 | 1848.17 | 0.96 | 1378.90 | 127.03 | 8.85 |
| CBB1 | 1216.58 | 0.98 | 899.80 | 89.19 | 8.08 |
| CBB2 | 1175.20 | 0.98 | 882.80 | 87.32 | 7.91 |
| CBB3 | 1234.61 | 0.97 | 936.60 | 92.99 | 8.13 |

**Table S1** Number of sequences analysed, observed diversity richness (OTUs) and diversity/richness indices of the 16S rRNA bacterial libraries obtained for clustering at 97% identity.

| **Length(bp)** | **Sequences** |
| --- | --- |
| 0-200 | 0 |
| 200-260 | 216 |
| 260-320 | 1764 |
| 320-360 | 3 |
| 360-380 | 14 |
| 380-400 | 1211 |
| 400-420 | 345393 |
| 420-440 | 260186 |
| 440-460 | 594 |
| 460-480 | 9 |
| 480-500 | 1 |
| 500-520 | 0 |
| 520-540 | 2 |
| 540-560 | 0 |
| 560-600 | 0 |

**Table S2** Quantitative distribution of high quality sequence length obtained through high-throughput sequencing.

**
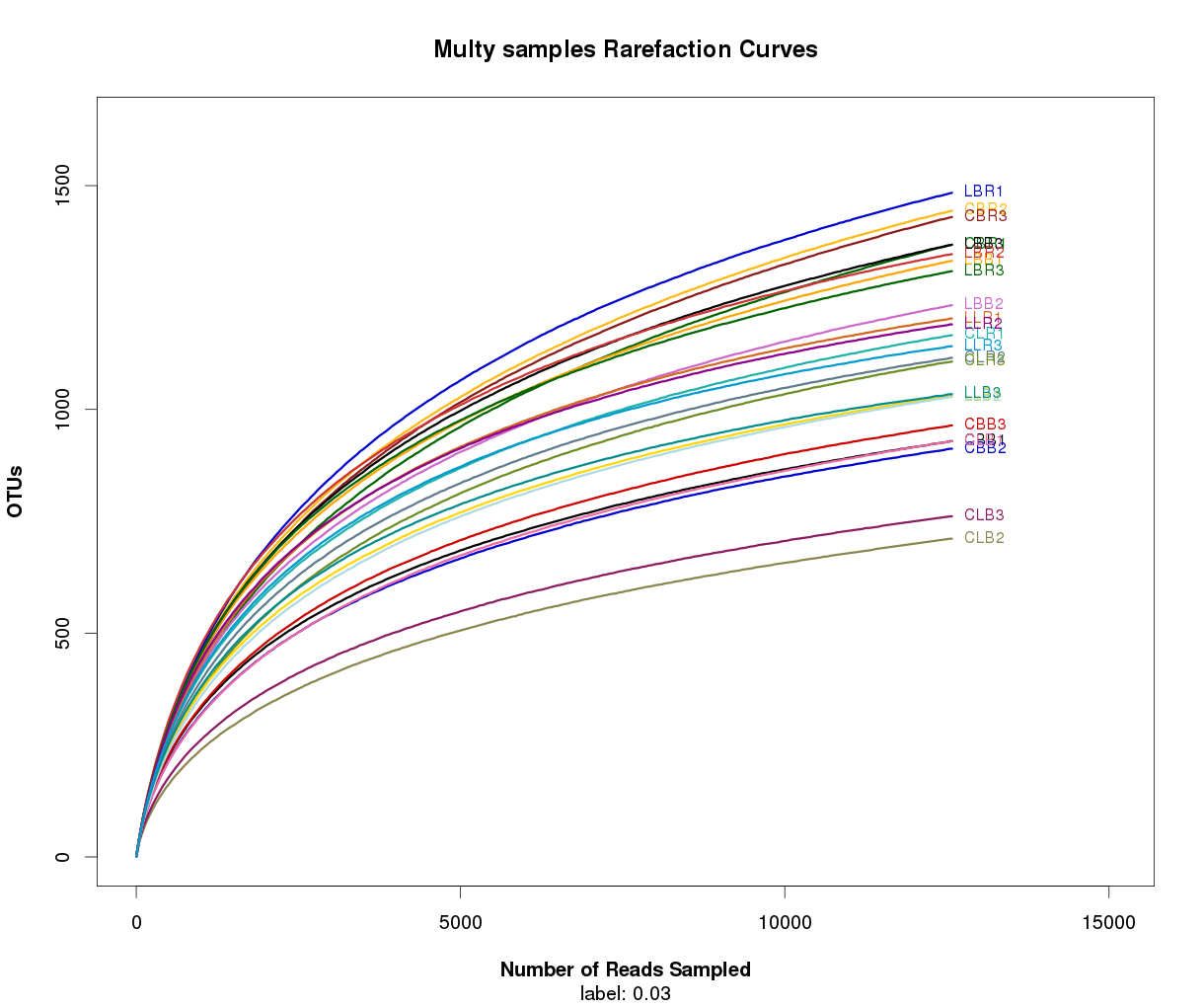
**

**Figure S1.** Multy samples rarefaction curves of bacteria depicting the effects of 3% dissimilarity on the number of OTUs identified in all samples. Samples were encoded with letters indicating their collection location (L, Lianmeng; C, Changlongjie), tree species (L, *L*. *lucidum*;B, *B*. *papyrifera*), substrate (R, rhizosphere; B, bulk soil) and number of replications (1-3).


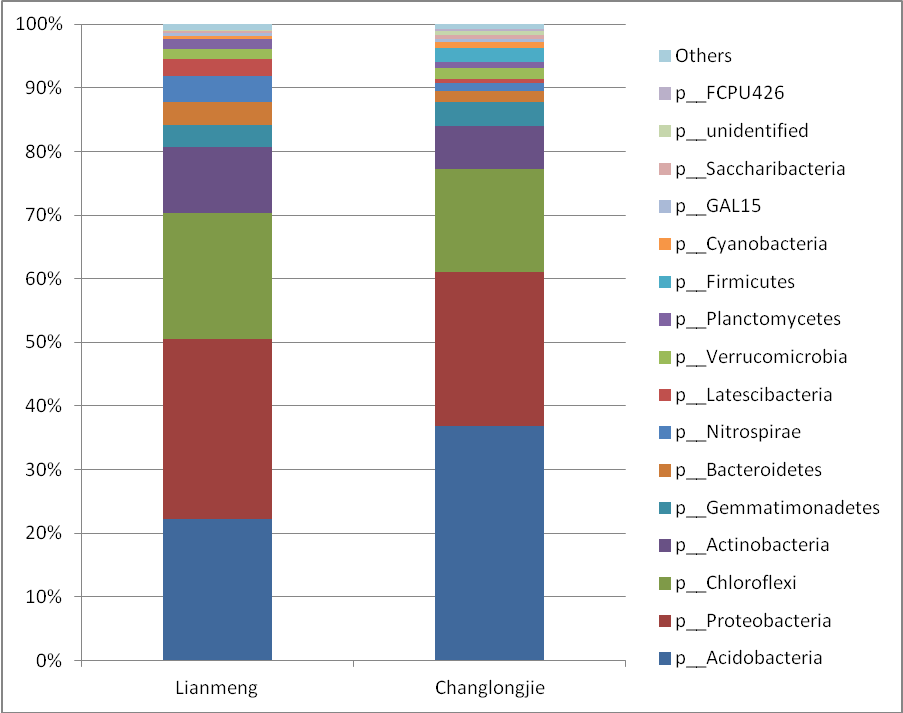


**Figure S2**. Relative abundance of bacteria at the phylum level in combined Lianmeng samples and combined Changlongjie samples.

Axis 2

11.85%
